# Genitourinary Tuberculosis in a Young Male With Advanced Human Immunodeficiency Virus (HIV) Diagnosed via Urinary Lipoarabinomannan Antigen (TB-LAM): A Case Report From a High-Burden Setting

**DOI:** 10.7759/cureus.95965

**Published:** 2025-11-02

**Authors:** Juan M Rodriguez Marino, Melanie Caballero Garcia, Alejandra Vargas, Brigida Tueros, Dhayana S Pelaez Callalli, Brillins S Pelaez Callalli, Fiorella Rodriguez

**Affiliations:** 1 General Medicine, San Martín de Porres University, Lima, PER; 2 General Medicine, Universidad Privada San Juan Bautista, Lima, PER; 3 General Medicine, Universidad Nacional Federico Villareal, Lima, PER; 4 School of Medicine, Universidad Norbert Wiener, Lima, PER; 5 Facultad de Medicina, Universidad de Aquino Bolivia, Santa Cruz, BOL

**Keywords:** diagnostic limitations, extrapulmonary tuberculosis (eptb), genitourinary tb, hiv aids, immunocompromised patient, lipoarabinomannan antigen, urine tests

## Abstract

Genitourinary tuberculosis (GU-TB) is a rare but significant manifestation of extrapulmonary tuberculosis, particularly among immunocompromised individuals. Diagnosis remains difficult in resource-limited settings where access to culture or advanced imaging is restricted. The World Health Organization recently endorsed the urinary lipoarabinomannan (TB-LAM) assay for early tuberculosis detection in people with advanced HIV.

We report the case of a 29-year-old mestizo man from Peru with HIV diagnosed in 2018 who had discontinued antiretroviral therapy (ART) shortly after initiation. He reengaged with the Peruvian healthcare system in July 2025, presenting with persistent systemic symptoms, including night sweats, anorexia, and lower abdominal discomfort. Laboratory studies revealed a CD4 count of 237 cells/μL and a viral load of 1.59 million copies/mL. Urinalysis showed sterile pyuria and microscopic hematuria. Chest radiography revealed no pulmonary involvement. Renal ultrasound demonstrated increased parenchymal echogenicity with partial loss of corticomedullary differentiation. Given his immunosuppressed state and nonspecific presentation, a TB-LAM test was performed and returned positive.

A diagnosis of GU-TB was established, and the patient was initiated on first-line anti-tuberculosis therapy with concurrent ART reintroduction. He demonstrated a favorable clinical response without immune reconstitution inflammatory syndrome (IRIS).

This case highlights the diagnostic utility of TB-LAM in extrapulmonary TB among HIV-positive patients and underscores the need for clinical vigilance in high-burden, low-resource settings.

## Introduction

Extrapulmonary tuberculosis (epTB) refers to a form of tuberculosis (TB) that involves sites outside the lungs [1]. Among its clinical variants, genitourinary tuberculosis (GU-TB) is particularly relevant, affecting the kidneys, ureters, bladder, and both male and female genital organs [2].

According to the 2024 World Health Organization (WHO) report, an estimated 10.8 million TB cases were documented, with extrapulmonary manifestations accounting for 16% [1]. In individuals with Human Immunodeficiency Virus (HIV) infection, the prevalence of epTB increases to nearly 50%, and within this subgroup, approximately 20% correspond to GU-TB [2].

GU-TB typically arises from hematogenous dissemination of a primary pulmonary focus. The chronic localized form is the most common presentation, characterized by insidious urogenital symptoms and progressive damage to the urinary tract. In contrast, the acute systemic form is uncommon and presents with generalized manifestations, disseminated disease, and renal parenchymal involvement with abscess formation [3].

The acute systemic form is most frequently observed in patients with HIV or other immunocompromising conditions [3]. The degree of immune suppression plays a critical role in disease progression, while microbiological confirmation remains difficult [1]. Due to its nonspecific clinical features and overlap with other conditions, GU-TB is frequently underdiagnosed, contributing substantially to morbidity, mortality, and reduced quality of life [4].

The purpose of this case report is to underscore the diagnostic value of the TB-LAM assay in extrapulmonary tuberculosis among HIV-positive patients and to stress the importance of sustained clinical vigilance in high-burden, resource-limited settings.

## Case presentation

A 29-year-old mestizo male from a low-income urban area in Peru presented to the infectious disease service with a one-year history of recurrent constitutional symptoms that had become persistent during the two weeks prior to evaluation. He reported night sweats, anorexia, mild lower abdominal discomfort, and fatigue. He denied fever, cough, dyspnea, diarrhea, or dysuria.

The patient had been diagnosed with HIV infection seven years ago, but discontinued antiretroviral therapy (ART) shortly after initiation. He reported completing six months of isoniazid preventive therapy at the time of diagnosis, but had not sought further medical care until the current presentation. He was classified as an ART defaulter and had recently reinitiated treatment with a tenofovir-lamivudine-dolutegravir (TLD) regimen, being on day seven of therapy at the time of evaluation.

**Figure 1:**
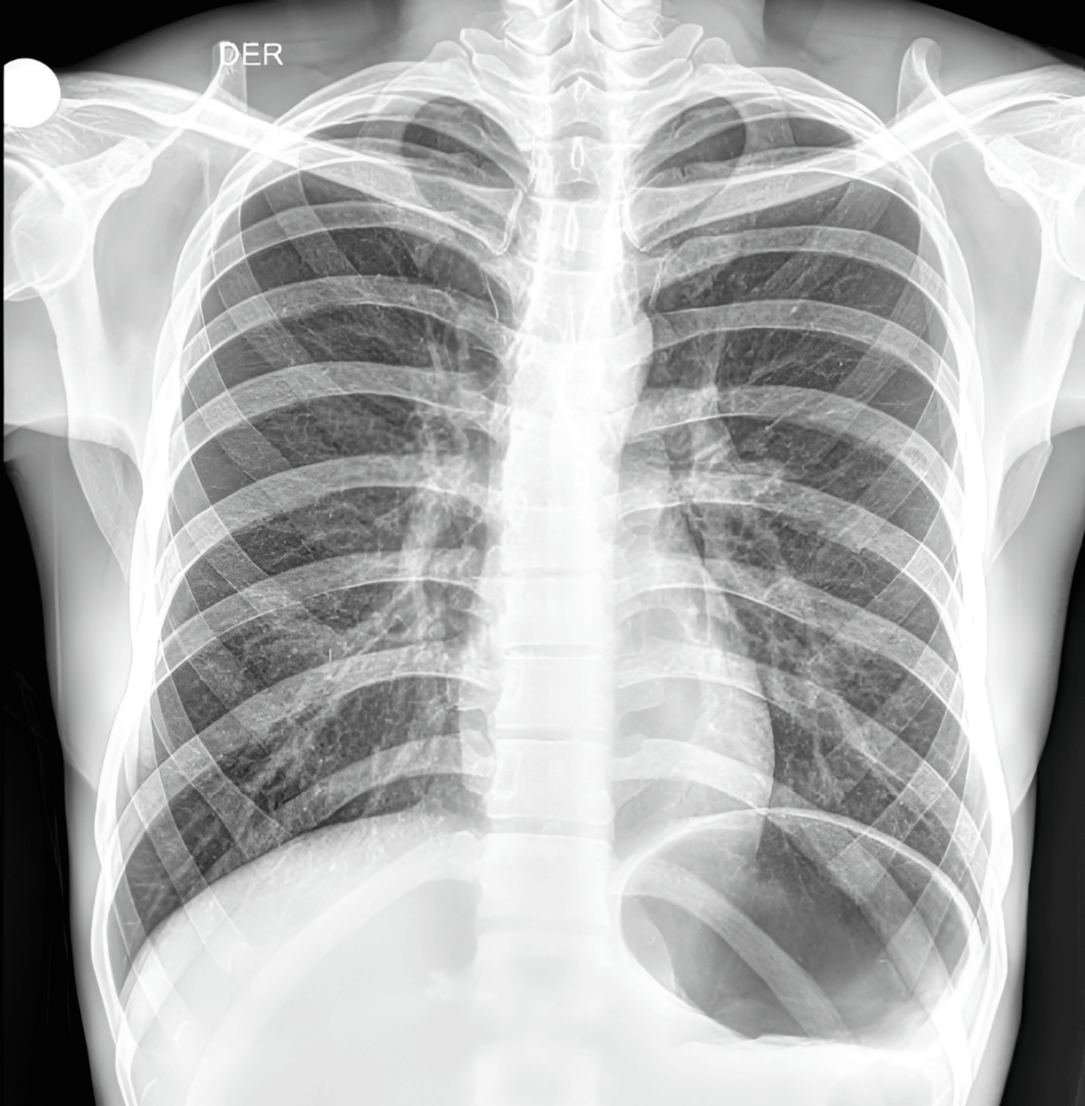
Chest X-ray, posteroanterior (PA) view Posteroanterior chest X-ray demonstrating clear lung fields without infiltrates, cavitations, or mediastinal lymphadenopathy.

On examination, he appeared chronically ill but was hemodynamically stable and in no respiratory distress. No lymphadenopathy, oral candidiasis, or organomegaly was noted. Vital signs were within normal limits. Abdominal examination revealed a soft abdomen with mild suprapubic tenderness, without costovertebral angle tenderness. Chest X-ray without alterations (Figure 1).

Laboratory studies showed anemia, hypoalbuminemia, elevated total protein, and mild elevation of alkaline phosphatase, while liver enzymes were within normal limits. HIV viral load was markedly elevated, with reduced CD4 count and inversion of the CD4/CD8 ratio (Table 1).

**Table 1:** Laboratory findings. AST: aspartate aminotransferase; ALT: alanine transaminase; HIV: human immunodeficiency virus; CD4: clusters of differentiation 4; CD8: clusters of differentiation 8.

Parameter	Patient Value	Reference Range
Hemoglobin	10.2 g/dL	13.5–17.5 g/dL
Serum creatinine	1.11 mg/dL	0.7–1.3 mg/dL
Total protein	8.44 g/dL	6.0–8.0 g/dL
Albumin	3.21 g/dL	3.5–5.0 g/dL
Alkaline phosphatase	145 U/L	44–147 U/L
AST	26 U/L	10–40 U/L
ALT	33 U/L	7–56 U/L
HIV viral load	1,590,000 copies/mL (log 6.20)	<40 copies/mL
CD4 count	237 cells/μL	500–1500 cells/μL
CD4/CD8 ratio	0.10	1.0–3.0
White blood cells (urine)	12–15 /hpf	0–5 /hpf
Red blood cells (urine)	1–3 /hpf	0–2 /hpf
Epithelial cells (urine)	2–3 /hpf	0–1 /hpf
Bacteria (urine)	None detected	Negative
Casts (urine)	None detected	Negative

Urinalysis showed 15-20 WBCs/hpf and 5-10 RBCs/hpf, with no bacterial growth on culture. Urine culture could not be performed due to resource limitations (Table 1).

**Figure 2:**
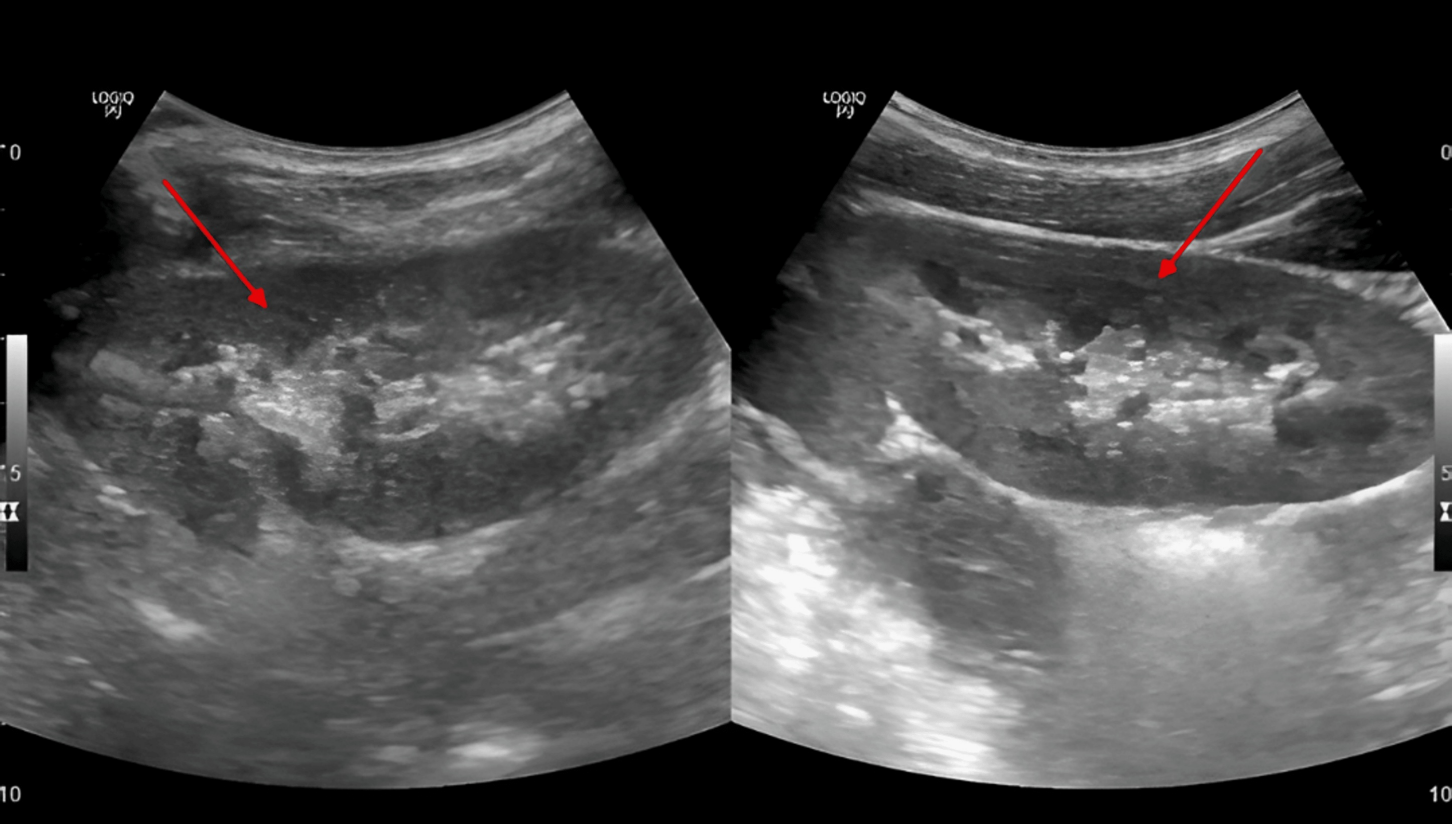
Renal ultrasound Revealed increased parenchymal echogenicity and partial loss of corticomedullary differentiation in both kidneys, findings suggestive of chronic renal inflammation or granulomatous disease

Given the patient’s immunocompromised state and high clinical suspicion for extrapulmonary tuberculosis, a urinary lipoarabinomannan (TB-LAM) antigen test was performed and returned positive. Based on the combination of sterile pyuria, abnormal renal ultrasound findings, positive TB-LAM, and absence of pulmonary involvement, a diagnosis of genitourinary tuberculosis was established (Figure 2).

The patient was initiated on first-line anti-tuberculous therapy (isoniazid 300 mg, rifampin 600 mg, pyrazinamide 1600 mg, and ethambutol 1200 mg daily for two months) with continuation of ART. Clinical evolution was favorable with the gradual resolution of systemic symptoms. The patient remained under close outpatient follow-up and exhibited no signs of immune reconstitution inflammatory syndrome (IRIS).

At the three-month follow-up, the patient had already completed two months of the first phase of antituberculosis therapy and had transitioned to the second phase with isoniazid (300 mg) and rifampin (600 mg). He exhibited significant clinical improvement, including resolution of dysuria and flank pain, a weight gain of 3.5 kg, recovery of renal function, and normalization of inflammatory markers, findings consistent with a favorable short-term response. His CD4 count increased from 237 to 312 cells/μL following ART reintroduction. Despite these encouraging outcomes, the long-term prognosis remains guarded due to his advanced immunosuppression; however, adherence to both antituberculous therapy and ART is expected to mitigate the risk of complications associated with the coexistence of tuberculosis and HIV infection.

## Discussion

Genitourinary tuberculosis (GUTB) represents the second most common form of extrapulmonary TB, often presenting with vague urinary symptoms that mimic other conditions. Diagnosis relies on a combination of microbiological, molecular, histological, and imaging techniques, yet clinical suspicion is frequently delayed due to its nonspecific presentation. Timely initiation of the standard six-month anti-TB regimen is essential to prevent irreversible urogenital damage [5]. This case exemplifies these challenges, as the patient experienced prolonged constitutional symptoms and sterile pyuria before the diagnosis was confirmed. Objective follow-up data, including a 3.5 kg weight gain and CD4 recovery from 237 to 312 cells/μL, support the favorable clinical response observed after initiation of antituberculous therapy and ART reintroduction.

Extrapulmonary tuberculosis represents 15-20% of TB cases, often mimicking other diseases and complicating timely diagnosis. The absence of pulmonary involvement in our patient further delayed suspicion, underscoring how atypical presentations can hinder recognition in immunocompromised hosts [6,7].

The diagnostic process was prolonged in this case, with the patient initially seen by several providers who did not suspect TB. The turning point occurred when a clinician, considering the persistence of atypical urinary symptoms and the lack of improvement with standard approaches, broadened the differential and pursued specific testing, which confirmed GUTB [8]. In Peru, TB-LAM is not universally available, which highlights inequities in diagnostic access and further emphasizes the need for investment in diagnostic capacity [9].

The 2023 World Health Organization (WHO) guidelines recommend the use of the urinary lipoarabinomannan (TB‑LAM) test in patients with HIV and CD4 counts below 200 cells/μL or who are seriously ill, even if unable to produce sputum [10]. Recent studies have shown that TB‑LAM improves TB detection and reduces mortality in hospitalized HIV-positive patients with low CD4 counts, particularly in Africa and Latin America [11,12]. In our case, although the CD4 count was slightly above 200 (237 cells/μL), the clinical profile warranted testing, and the positive result facilitated early diagnosis.

In extrapulmonary TB, imaging is essential for detection and monitoring but must be integrated with microbiological or histological assays to ensure diagnostic accuracy [6]. The integration of imaging with laboratory-based assays is therefore critical. In this case, the urinary lipoarabinomannan (TB-LAM) antigen test was decisive. Although its sensitivity is limited in patients with higher CD4 counts, TB-LAM remains a valuable diagnostic tool for TB in people living with HIV and advanced immunosuppression [7].

The sensitivity of AlereLAM ranges between 45% and 56% in patients with CD4 <200 cells/μL, with a specificity around 90% [13]. Meanwhile, FujiLAM, a newer generation test, has shown sensitivity rates of 67-70% and specificity of 90-99% in hospitalized patients with advanced HIV [14]. A recent review highlighted the diagnostic yield of TB-LAM when used alongside molecular tests like GeneXpert and mycobacterial culture, especially in extrapulmonary TB [15].

Beyond the clinical dimension, this case also reflects broader systemic issues in TB control in Peru. Qualitative and mixed-methods research has revealed that barriers such as inadequate training, poor integration between HIV and TB programs, and stigma contribute to diagnostic delays [7]. Furthermore, studies in Peruvian hospitals have shown that knowledge of TB among HIV patients remains limited, which may reduce healthcare-seeking behavior and adherence to treatment [16]. These gaps emphasize the need not only for better diagnostic tools but also for ongoing provider education and community-based interventions.

Similar cases reinforce the protean nature of GUTB. For example, testicular TB mimicking malignancy has been reported, often resulting in unnecessary orchiectomy before the correct diagnosis is established [17]. Likewise, renal TB has been described in adolescents presenting with hydronephrosis, underscoring its ability to imitate other urologic conditions [18]. These cases, together with ours, highlight the diagnostic pitfalls of GUTB and the importance of considering TB in the differential diagnosis of unexplained genitourinary disease, particularly in endemic settings.

Genitourinary tuberculosis exemplifies the diagnostic pitfalls of extrapulmonary TB, where nonspecific urinary symptoms, absence of pulmonary involvement, and systemic barriers in care contribute to delays. Integrating imaging with microbiological and molecular assays and incorporating tools such as TB-LAM can improve diagnostic yield, particularly in immunocompromised populations in endemic regions [1-18].

## Conclusions

Genitourinary tuberculosis represents a form of extrapulmonary tuberculosis whose nonspecific clinical presentation often delays diagnosis, particularly in resource-limited settings. This report highlights the importance of maintaining a high index of suspicion in immunocompromised patients, such as those with HIV infection, in whom timely diagnosis is essential to reduce complications and improve clinical outcomes.

In this context, considering genitourinary tuberculosis in patients with persistent constitutional or urinary symptoms, even in the absence of pulmonary involvement, is crucial. Recognition of sterile pyuria, careful interpretation of imaging findings, and the use of rapid assays such as TB-LAM as a supportive rather than definitive diagnostic tool enhance diagnostic accuracy and allow for early initiation of treatment. Nevertheless, the diagnostic process remains limited by restricted access to confirmatory molecular and culture-based tests in many low-resource settings, which may delay or obscure diagnosis. Expanding access to point-of-care diagnostics, effectively integrating TB and HIV programs, and strengthening healthcare provider training are therefore essential priorities. Additionally, future research on novel assays such as FujiLAM could further optimize early detection and clinical outcomes in highly vulnerable populations.
